# Setting research priorities to reduce malaria burden in a post graduate training programme: lessons learnt from the Nigeria field epidemiology and laboratory training programme scientific workshop

**DOI:** 10.11604/pamj.2014.18.226.4800

**Published:** 2014-07-17

**Authors:** Olufunmilayo I Fawole, Olufemi Ajumobi, Gabriele Poggensee, Patrick Nguku

**Affiliations:** 1 Nigeria Field Epidemiology and Laboratory Training Programme, Abuja, Nigeria; 2 Epidemiology and Medical Statistics, Faculty of Public Health, College of Medicine, University of Ibadan, Nigeria; 3 National Malaria Elimination Programme, Abuja, Nigeria

**Keywords:** Setting Malaria Research Priorities, Post graduate Training Programme, Malaria workshop, Malaria Research, Post graduate students Research

## Abstract

Although several research groups within institutions in Nigeria have been involved in extensive malaria research, the link between the research community and policy formulation has not been optimal. The workshop aimed to assist post graduate students to identify knowledge gaps and to develop relevant Malaria-related research proposals in line with identified research priorities. A training needs assessment questionnaire was completed by 22 students two week prior to the workshop. Also, a one page concept letter was received from 40 residents. Thirty students were selected based the following six criteria: - answerability and ethics; efficacy and impact; deliverability, affordability; scalability, sustainability; health systems, partnership and community involvement; and equity in achieved disease burden reduction. The workshop was over a three day period. The participants at the workshop were 30 Nigeria Field Epidemiology and Laboratory Training Programme (NFELTP) residents from cohorts 4 and 5. Ten technical papers were presented by the experts from the academia, National Malaria Elimination (NMEP) Programme, NFELTP Faculty and Implementing partners including CDC/PMI. Draft proposals were developed and presented by the residents. The “strongest need” for training was on malaria prevention, followed by malaria diagnosis. Forty seven new research questions were generated, while the 19 developed by the NMEP were shared. Evaluation revealed that all (100%) students either “agreed” that the workshop objectives were met. Full proposals were developed by some of the residents. A debriefing meeting was held with the NMEP coordinator to discuss funding of the projects. Future collaborative partnership has developed as the residents have supported NMEP to develop a research protocol for a national evaluation. Research prioritization workshops are required in most training programmes to ensure that students embark on studies that address the research needs of their country and foster collaborative linkages.

## Introduction

Malaria is highly endemic in Nigeria and it remains one of the leading causes of childhood and maternal morbidity and mortality in the country [1]. High temperature, humidity, and rainfall are some of the factors that enhance mosquito breeding and malaria transmission [2]. Malaria accounts for 60% of outpatient visits, and for 30% of hospitalisations among children under five years of age [3, 4]. Childhood deaths resulting from malaria have been estimated at over 300,000 deaths per year in Nigeria [5]. At this rate, malaria accounts for more deaths per year than HIV/AIDS, and is the major contributor to deaths in children under-five years and in pregnant women [3]. The National Malaria Indicator Survey of 2010 showed that slide positivity rate was 42% in children under-five [5]. Pregnant women living in places where malaria is highly prevalent are four times more likely than other adults to get malaria and twice as likely to die of the disease [6]. Malaria related deaths account for up to 11% of maternal mortality, 25% of infant mortality, and 20% of under-five mortality resulting in 300,000 childhood deaths annually [3, 5]. The Federal Government of Nigeria is deeply committed to making progress towards the achievement of the Millennium Development Goals and it recognizes that, without firm efforts to control malaria, achievement of the targets related to child mortality, maternal mortality, and reducing the burden of communicable disease will not be possible [1]. Malaria therefore constitutes a significant development challenge for Nigeria. In 1998 the Roll Back Malaria (RBM) Partnership was launched in Nigeria as a dynamic movement involving all stakeholders affected by or concerned with Malaria. Although Nigeria is a signatory to the Abuja Declaration of year 2000, the agreed targets have not been achieved [1].

The 2009-2013 National Malaria Strategic Plan (NMSP) identifies operational research on malaria as crucial in informing continuous adjustment of policies and implementation strategies. Though several research groups within institutions in Nigeria have been involved in extensive malaria research for many years, the link between the research community and policy formulation has not been optimal. Many of the findings are neither shared with the National Malaria Elimination Programme (NMEP), nor disseminated internationally [3]. Also NMEP has prioritized a list of research priorities of which some researchers are unaware [1]. The Field Epidemiology and Laboratory Training Programme (FELTP) is a two year master's training in applied epidemiology. FELTP serves to build local capacity to improve and strengthen countries’ public health systems and infrastructure. The Nigeria FELTP commenced in 2008, and since then has been working to build capacity in the public health workforce [7]. Presently, the programme is in its sixth cohort of student intake (www.nigeria-feltp.net). With support from United States Presidents Emergency Plans for AIDs Relief (PEPFAR)) through the Centers for Disease Control and Prevention (CDC), Presidents Malaria Initiative (PMI), the NFELTP trains medical, laboratory and veterinary field epidemiologists. Prior to their training, the trainees or residents work in leadership and technical positions in the Federal Ministries of Health (FMOH) and Agriculture and Rural Development (FMARD), State Ministries and Local Government Health Departments including in Research Institutions. The program has helped to improve public health systems within the country by developing a cadre of well-trained and competent public health professionals [7].

The residents are expected to conduct research projects and write a dissertation as part of the requirements for award of the Master degree. They are also encouraged to participate in scientific conferences, write field reports and publish as part of their training. Hence, malaria-related research studies have been conducted by the graduates and trainees of the programme. A review of previous malaria projects of the residents admitted in the first two years of the programme showed that three students (11.5%) out of the 26 residents in the first two cohorts had done original research on malaria. These studies assessed case management practices and evaluated laboratory diagnostic tools and services. Other studies conducted by the residents on the programme have included evaluation of malaria surveillance systems at the different tiers of the health system and secondary data analyses of malaria databases [8]. However, there is a need to involve more residents in research on Malaria and expand the scope of these studies to include contemporary international, national and local issues. Also, there is the need to ensure that the studies conducted are addressing the research needs and agenda of the country. PMI is one of the funders of the program, therefore the residents work need to reflect PMI focus. Lastly, there is a crucial need for a comprehensive inventory of research areas that address the problems identified in malaria control in the country. This road map should address the research needs identified by the various stakeholders involved in Malaria control and elimination in Nigeria. Furthermore, the inventory will assist future NFELTP residents to identify topics for their research work.

**Workshop objectives and expected outcomes:** The objectives of the workshop were to assist residents to identify research areas on Malaria with knowledge gaps and to encourage and support NFELTP residents to develop relevant Malaria-related research. The expected outcomes were development of an inventory of Malaria-related research topics which can be addressed by NFELTP residents, development of draft study proposals and provision of networking opportunities for NFELTP residents and stakeholders.

## Methods

The implementation of the workshop was done in three stages, namely pre workshop, the workshop and post work shop activities.

### 1. Pre-Workshop

The pre workshop stage also had three main activities: - preparatory activities, training needs assessment and the submission of concept notes.

***Preparatory work:*** Preparatory to the workshop, the “NFELTP organization of a workshop checklist” was used as a guide [9]. The title of the workshop, a provisional date and responsible persons for organisation were selected. The questionnaire and concept note format were developed and sent to residents. The agenda, instruction for presenters and guideline for the group work activities were developed and shared with the facilitators. Hands-out and presentations were developed and printed. The presentation format for the group work and proposal writing guide were also developed and sent to the residents. Identification of Malaria experts- A list of potential facilitators was developed. The facilitators cut across the different sectors namely: - Representatives from the Federal Ministry of Health, Center for Disease Control (CDC), Roll Back Malaria (RBM) Implementing partners and academia. The selection of academics that made presentations was based on the themes of the concepts. The NFELTP faculty also played an important role during the workshop. Letters of invitation was sent to the selected experts.

**Figure 1:**
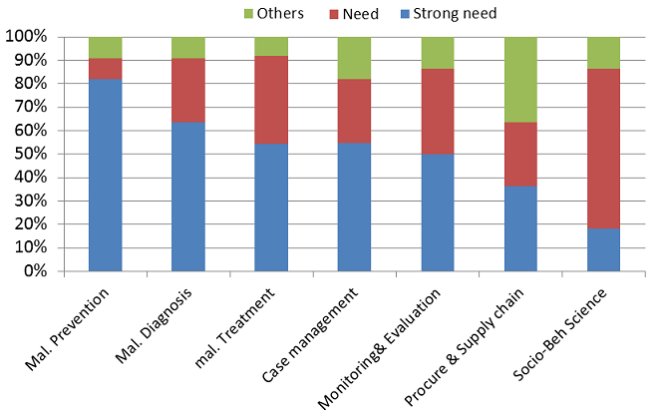
Training needs of the residents on general thematic areas of Malaria research others were informed but not decided, less need, no need and not sufficiently informed

***Training needs assessment:*** The purpose of the training needs assessment was to ensure the workshop addressed resident's needs. It was conducted from the 31st August to the 13th September, 2013. It had two components, a questionnaire survey and the submission of a concept letter. Between the 31st August and 6th September 2013 a structured self administered 63 item questionnaire was sent to 86 residents by electronic mail. The questionnaire had two main sections, a general and specific assessment. The degree of research needed in seven general areas was assessed using a 6 point Likert scale. Responses ranged from being “not sufficiently informed” on the subject matter to “strong need, need, undecided, less need, no need”. In the specific assessment sections seven areas were assessed namely: -Prevention, Diagnosis, Treatment, Procurement and Supply Chain Management, Case Management, Social and Behavioral Sciences, Monitoring and Evaluation. The areas requiring the most urgent attention on each of these specific thematic areas were asked with a series of questions ranging from 3 to 10 per theme. The responses were scored as follows - “strong need” -2 marks, “need” - 1 mark. Persons who were undecided or who indicated less or no need scored no marks, while those who stated that they were not sufficiently informed were excluded from the scoring (Table 1). Twenty two residents responded to the needs assessment hence non response rate was 25.6%. On the general areas in which research are strongly needed, most residents indicated “malaria prevention”, followed by “malaria treatment” and then “malaria diagnosis ”(Figure 1). As regards the specific themes requiring most urgent research on malaria prevention most of the residents indicated “vector control”, that is, “uptake of insecticidal treated nets (ITN) and long lasting insecticidal nets (LLIN), followed by “maternal knowledge, attitude and practice and childhood malaria infection”. On “malaria treatment” the strongest need (50%) was indicated on “use of rapid diagnostic test (RDTs) and adherence to treatment guidelines in the public and private sector”. While on malaria diagnosis it was “quality control/assessment measures of microscopy and RDT and its effect on malaria diagnosis” (Table 2 (a), Table 2 (b)).

**Table 1:** Students assessment of training needs required on malaria research

Theme	Research questions in training need questionnaire	Not sufficiently informed(no;%	Average score
**Malaria prevention**	Determinants of Sulphadoxine-pyrimethamine (SP) uptake for intermittent preventive treatment in pregnancy (IPT)	1(4.5)	1.2
	Pregnant women's understanding and adherence to IPT Regimen	1(4.5)	1.3
	Maternal knowledge, attitude and practice and childhood malaria infection	2(9.1)	1.3
	Availability and access to IPT for pregnant women	0(0)	1.3
	Promotion of malaria preventive practices with the use of the present health promotion mediums (media, print)	0(0)	1.1
	Potentials of larviciding as part of the scale up of integrated vector management	2(9.1)	1.2
	Household size/composition effect on adherence to use of ITN	0(0)	1.2
	Evaluation of combination of prevention approaches - ITNs/IRS & Laviciding	2(9.1)	1.1
	The cost effectiveness of LLINs compared to IRS	2(9.1)	0.7
	Training for midwives to improve IPT uptake	0(0)	0.8
**Malaria diagnosis**	Quality control measures on laboratory diagnostic testing (microscopy and rapid testing)	0(0)	1.2
	Determinants of under-diagnosis and over-diagnosis of malaria in children under 5s	0(0)	1.2
	Cost and availability of diagnostic tests - microscopy and rapid tests	0(0)	1.0
	Patent medicine vendors providing rapid diagnostic test	0(0)	1.0
**Malaria treatment**	Effect of HIV co-infection on treatment of malaria	1(4.5)	1.2
	Barriers to treatment seeking for children under 5	0(0)	0.5
	Effect of knowledge and attitude on choice of care for malaria	0(0)	1.2
	Providers’ behavior and perceptions on the new malaria treatment guidelines	0(0)	1.2
	Knowledge and practices of health care workers on the new malaria treatment policy	0(0)	1.1
	Barriers to compliance with administered prescriptions	0(0)	1.1
	Direct and indirect costs associated with malaria treatments	0(0)	1.1
	Adherence to treatment guidelines in the public and private sectors	0(0)	1.5
	Factors contributing to the poor uptake of ACTs	0(0)	1.3
	Availability, accessibility and affordability of ACT therapy	0(0)	1.2
	Maternal knowledge and practice influence on burden of malaria in children	0(0)	1.2
**Procurement & supply**	Stock outs and its effect on malaria management.	0(0)	1.1
	Best approaches for ensuring uninterrupted supplies of quality-assured ACTs, LLINs and RDTs	1(4.5)	1.1
	Understanding the market of patent medicine vendors (PMVs)	0(0)	1.0
	Implications of over-diagnosis and overtreatment on the supply and availability of ACTs	0(0)	0.9
	Use of 3^rd^ party logistic agents (3PLS) improve the promptness of distribution of malaria commodities	3(13.5)	0.6
	Sustainable and predictable financing for adequate forecasting and uninterrupted flow in the malaria management	1(4.5)	0.4
**Case management**	Determinants of artemisin-based combination therapy access and use	1(4.5)	1.0
	Health system factors that limit case management of malaria in pregnancy	0(0)	1.1
	Geographical, economic and social issues affecting malaria management	0(0)	1.0
	Evaluating proposed amount of ITN/LLITN provided per family	1(4.5)	1.0
	Consequences of negative RDT/microscopy result in management of febrile illnesses	0(0)	1.4
	Training of health care providers and effect of adherence to treatment by users	1(4.5)	1.4
	Appropriate subsidy levels and relative pricing of ACTs and RDTs	0(0)	1.0
	Training of trainers (TOT) to scale-up malaria management	0(0)	1.0
**Socio-behavioral**	Effect on health seeking behaviors and prompt diagnosis and treatment	1(4.5)	1.1
	Community involvement in recognizing malaria incidences and improving health-seeking behaviors	0(0)	1.0
	Link between socioeconomic status of caregivers and malarial outcomes on mothers and child	1(4.5)	1.1
**Monitoring & evaluation**	Causes of poor and inaccurate data reporting in Nigeria	0(0)	1.4
	SMS applications and DHIS to improve the data quality and feedback	0(0)	1.1
	Surveillance systems and its effectiveness in tracking insecticide and drug resistances	0(0)	1.4
	Record-keeping measures in health facilities and its adequacy	0(0)	1.2
	Evaluating the use and upkeep of the LLINs	1(4.5)	1.0

**Table 2 - a:** Themes of Concept Letters Received from Residents for the Malaria Workshop

Theme	Cohort	Track	Title/Research Questions	Scores	Percent
**Malaria in Pregnancy**				-44	
1	5	Lab	Malaria parasitaemia and risk factors of Malaria among pregnant women attending antenatal care in Nasarawa South LGA of Nasarawa State	29	66
2	5	Lab	Assessment of the impact of malarial intermittent preventive therapy on maternal parasitaemia at delivery	28	64
3	5	Med	Assessment of malaria preventive practices amongst pregnant women attending antenatal care services, Osogbo, Osun State	30	68
4	5	Lab	Evaluation of intermittent preventive therapy (sulphadoxine pyrimethamine) uptake, at the antenatal clinic of University of Benin Teaching Hospital, Benin	26	59
5	5	Med	Knowledge and utilization of intermittent preventive treatment for malaria among pregnant women attending antenatal clinic in primary health centers in Akure, Ondo State.	31	70
6	5	Med	What health systems factors limit case management of malaria in pregnancy in urban and rural settings in Zamfara State	32	73
**Vector Control**					
7	5	Lab	Malaria vector control practices in an irrigated rice agro-ecosystem in Nasarawa State, North central Nigeria and implications for malaria control.	33	75
8	4	Med	Comparison of prevalence of malaria parasitaemia between an indoor residual sprayed community and a non-indoor residual sprayed community in Nasarawa Eggon LGA of Nasarawa state	28	64
9	5	Vet	Malaria control using permethrin applied to tents of Nomardic Fulanis of North West Nigeria	30	68
**Prevention-Nets**					
10	5	Vet	Knowledge, attitudes and practices of ITN AND LLIN use among mothers of under -five children in Abakaliki, Ebonyi State	31	70
11	4	Lab	Effect of non-adherence to the use of mosquito bed nets on malaria disease burden in Makama Community, Lafia, Nasarawa, State, Nigeria	32	73
12	5	Med	Factors influencing adherence to the use of long lasting insecticide treated nets among under five children in Osogbo, Osun State	31	70

**Table 2 - b:** Themes of Concept Letters Received from Residents for the Malaria Workshop

**Laboratory Methods**					
13	4	Lab	Malaria infection among children less than 5 years living with HIV attending ART clinic at selected health facilities in Jos, Nigeria	29	66
14	4	Lab	Prevalence and risk factors of malarial parasitaemia among blood donors in Minna, Niger State	33	75
15	5	Lab	Quality control measures influencing accuracy of malaria rapid diagnostic test in Kano State	32	73
16	5	Lab	What are the trainings available to medical lab scientists on malaria diagnosis in a Lassa-endemic State (Edo State)?	30	68
17	4	Lab	Validation of cyscope microscope quantitative buffy coat and rapid diagnostic kit for malaria diagnosis among clinic attendees in University College Hospital Ibadan	29	66
18	5	Lab	What factors influence the use of rapid diagnostic tests and adherence to treatment guidelines in the public and private sectors?	33	75
19	5	Lab	External quality assurance of malaria diagnosis (microscopy and mRDT) in Kano State	31	70
**Economics of Malaria**					
20	4	Med	Household costs of malaria treatment in children under five years olds in Lafia LGA Nasarawa State	36	80
21	5	Med	Cost effectiveness analysis of indoor residual spraying and long lasting insecticide nets in Rivers State, South-South, Nigeria.	36	80
**Case Management**					
22	5	Med	Factors influencing the use of rapid diagnostic tests and adherence to treatment among health care providers in the public and private sectors in Ogun State	33	75
23	4	Med	Factors associated with adherence to guidelines on treatment of malaria among physicians at Federal Medical Centre, Lokoja, Kogi State, Nigeria	28	64
24	5	Epid	Factors associated with ACT treatment failure among adults in Bauchi State	31	70
25	5	Epid	Risk factors for severe malaria in children underfives in Maternal and Child Health Hospital, Gusau	28	64
26	5	Med	What is the effect of HIV co-infection on the treatment of malaria in children under five?	29	66
**Socio Behavioural**					
27	5	Med	Knowledge and practices of primary health care workers and care givers on the use of ACT for malaria treatment in under-fives in Osogbo, Osun State	34	77
28	5	Med	Knowledge, attitude, beliefs and practices of trained and untrained patent medicine vendors on case management of malaria in Kano state	32	73
29	5	Med	Role of patient medicine vendors in the treatment of malaria in Oyo State	28	64
30	5	Med	Knowledge and practices of health care workers on the new malaria treatment policy	34	77

***Development of concepts:*** Based on the specific questions on the thematic areas, the students submitted one page concept notes to address any of the needs identified. Concepts consisted of an introduction, research question, methodology and expected outcomes. Forty concepts notes were received from which 30 were selected. Selection was based on the following six criteria: - answerability and ethics (5 questions); efficacy and impact (3); deliverability, affordability, scalability, sustainability (6); health systems, partnership and community involvement (4); and equity in achieved disease burden reduction (4). Each of these criteria was scored 0, 1 or 2 points based on response to a series of questions that addressed these issues. Scores were summarized for each concept; the maximum score obtainable was 44, while the minimum was 0. Scores were then converted into percentage (Table 2 (a), Table 2 (b)). The scoring was done by two experts who were NFELTP faculty. Analysis of the concept letters showed that 7(23.3%) were from students in cohort 4 and 23 (76.7%) cohort 5 residents. Sixteen students were from the medical (53.3-%), two (6.7-%) from the veterinary and 12 (40.0%) from the laboratory tracks (Table 3). Most of the concepts (9/30) addressed “Prevention of Malaria” although there was overlap in themes of the concept letters. None was on “Monitoring and Evaluation” (0/30).

**Table 3:** Technical presentations at NFELTP Malaria Workshop - Strengthening Malaria-related research of NFELTP

	Title	Facilitator
1	Objectives and expected outcome of workshop	NFELTP porgramme
	Results of training needs assessment	
2	Highlights of research accomplished in the NFELTP	NFELTP programme
3	National malaria elimination programme (NMEP) priority research question	NMEP
4	The National malaria indicator survey	NMEP
s5	The National malaria control policies and guidelines	NMEP
6	What are the national knowledge gaps and relevant research questions - Case management and malaria in pregnancy	Academia
7	What are the national knowledge gaps and relevant research questions - - Community management	Academia
	- Role of the public /private health sectors	
8	What are the national knowledge gaps and relevant research questions - Vector control	Academia
9	What are the national knowledge gaps and relevant research questions - Laboratory diagnostics	Academia
10	What are the national knowledge gaps and relevant research questions - Socio behavioural aspects of control	Academia
11	What are the national knowledge gaps and relevant research questions - Economics of malaria control	Academia
12	What are the national knowledge gaps and relevant research questions	Academia
	– Surveillance	

### 2. The Workshop

The workshop was held between the 23rd and 25th of September, 2013. The participants were selected NFELTP residents from cohort 4 and cohort 5 (30); Federal Ministry of Health (1); representatives from the National Malaria Elimination Programme (NMEP) (2); facilitators from CDC Atlanta and Nigeria (2); PMI /USAID (1), and other RBM Implementing partners (2); Academia - University of Ibadan and Ahmadu Bello University Zaria (6); NFELTP Faculty (8). The workshop was primarily targeted at cohort 4 and cohort 5 NFELTP residents. These are students in their second and first year of training respectively. The implementing partners were encouraged to also send their interns and new staff for the workshop.

***Opening:*** Welcome remarks were given by the representative of the national coordinator of the NMEP. The gathering was reassured that there was political will and support for malaria elimination in Nigeria. The participants were also reminded the importance of operational research in guiding policy and program performance, and informed that the outcome of the deliberations would be useful in the forthcoming strategic plan review. The director of the programme, asked the experts to identify the knowledge gaps and enhance the skills of the residents through applied research, while the resident advisor encouraged the residents to be ready to work hard to get the best from the workshop.

***Technical presentations:*** As shown in Table 3, 10 papers were presented. There was also a remote presentation from CDC Atlanta, USA. The facilitators/presenters were representatives from the National Malaria Elimination Programme (NMEP), Nigeria CDC, and other Roll Back Malaria partners (US Malaria Presidential Initiative/Malaria Action Program for States, Society for Family Health, United States Agency for International Development, academia, and NFELTP Faculty. On the first day the knowledge gaps on the thematic areas was presented by experts from the National Malaria Elimination Programme and the academia (Table 3). The former presented the results of the National Malaria Indicator Survey and the highlights on the malaria treatment policies and guidelines, while the academics presented the knowledge gaps on the concept themes (Table 3). The concept letters developed by the residents were shared with the facilitators in preparation for the group activity the following day. The second and third days were strictly for group work and presentation of group work activities by the students. Four groups were constituted. The group work activities consisted of development of a draft proposal by the students and review of proposal by experts. The students presented their improved drafts at plenary sessions. Copies of the report on the “National Malaria Indicator Survey” were given to all the participants.

***Inventory of malaria research questions:*** An Inventory of research questions was generated. The NMEP shared the 19 questions which they had earlier developed in 2012. The questions from the NMEP were on the following themes:- case (5) management, integrated vector management (5), malaria in pregnancy (5) and cross cutting issues (4). The expert from the universities provided the following research questions:- Case management and malaria in pregnancy, two; community management, six; role of public and private health sectors, six; vector control, eight; laboratory diagnostics, eight; socio-behavioural aspects of control, 14; economics of malaria control, eight; surveillance, three.

***Development of drafts proposal:*** All residents commenced development of their proposals. Fifteen residents presented drafts of their proposal as power point presentations for input from the academic and programmatic experts.

***Evaluation:*** The evaluation form was filled by 10 participants. All (100%) either “strongly agreed or agreed” that the workshop objectives were met and overall the workshop was satisfactory. The subject matter was found to be useful to their dissertation or training. All also either strongly agreed or agreed that adequate time was allotted for explanations/group work and the presentations/materials provided was satisfactory. The group work was stated by 50% as what they liked most about the workshop followed by the presentation of the facilitators (40%). What was liked least about the workshop was that some facilitators were not fully available for the group work (10%), some said it was the lectures given (10%), while many (80%) had no response. If the workshop was repeated, what should be changed were as follows:-10% said it should be extended to five days, 10% said allocate more time for group work while, 20% wanted all the resource materials to be made available before the workshop commences.

### 3. Post workshop Next Steps/Follow-Up

At the end of the workshop it was agreed that a revised draft proposal was sent to the workshop coordinators and supervisors three weeks later. A debriefing meeting was held with the NMEP to advocate for funds to support the projects and foster collaborative linkages. Feedbacks were given to residents that submitted proposals. Some of these residents have written these up, as full proposals for their university thesis. Collaborative partnership began six months after the workshop as NFELTP residents and trainers supported NMEP to develop a research protocol for evaluation of Global Fund-supported training of health workers on malaria intervention. This will be implemented in 900 health facilities with interview of 900 health facility staff, 1800 direct clinical observations, 1800 client exit interviews patients across 12 states in Nigeria.

## Discussion

The fact that malaria still constitutes a public health challenge that is high on the research agenda of the country was evident at the workshop. Malaria accounts for more cases and deaths in Nigeria than any other country in the world, affecting 97% of its population. Persons most vulnerable to malaria and its complications are children under five years and pregnant women [10]. Thus Malaria is a topical issue for researchers including post graduate students to work on [11]. This was obvious at the programme, as prior to the workshop some students had already conducted field investigations on the disease. These studies included primary surveys and secondary data analysis [8]. Also many submitted concept notes suggesting interest in malaria research. Majority of the residents who sent concepts were the students in their first year of training. These are students who were trying to identify research topics for their dissertation and took the opportunities provided at the workshop to do so. Most of the students in the second year had already commenced their research work on different public health problems and were in various stages of implementation (hence our relatively high non response rate). However, those who had ongoing malaria research projects seized the opportunity to fine-tune their proposals with expert guidance. The timing of the workshop was also beneficial to first year residents who were in the nurturing process of developing research ideas for their university thesis. Overall, the workshop provided an opportunity to develop high quality research proposals because of the rigour involved in preparation of university dissertation that is of public health relevance and policy implementation in Nigeria. From the training needs assessment many students indicated a “strong need or need” for training on malaria prevention, diagnosis and treatment. Many of the concept notes were on these aspects. Their interest in these areas may be because the main strategies for Malaria control in Nigeria are based on these strategies. Effective case management of malaria infection, use of insecticide treated nets (ITN), indoor residual spraying and intermittent preventive treatment (IPTp) for pregnant women are the recommended measures in the National guidelines on malaria control [12]. The concept notes submitted guided the selection of facilitators for the workshop. They did not indicate much need for training on “Procurement and supply chain management”. Unlike with the absence of concept notes on Monitoring and Evaluation and socio behavioral science the students indicated interest of these aspects of Malaria control.

The highest proportion of concept notes was on “prevention of malaria”, followed by “malaria treatment”. This is may be the thrust of the FELTP training is on disease prevention and control. The theme of the concept notes also reflected the students training tracts, most were in the medical track. There was some overlap in some of the FMOH themes namely -“prevention”,“ treatment”, “diagnosis” and “case management”, therefore for the group work exercises, the methodology and target population of the proposed study were taken into consideration. The students did not submit concepts on “procurement and supply chain management”. Also the students did not indicated interest for training on this aspect during the needs assessment. This may because they did not knowledge on this aspect of malaria control or the skills to conduct studies in this area. The workshop facilitators stimulated interest of the students in research on this aspect and currently, a student is now conducting a research study on Procurement and Supply Chain Management. Also students did not submit concepts on “Monitoring and Evaluation”, of which surveillance is a crucial aspect, probably because many were experienced in evaluating disease surveillance systems. But this might also be because, developing a research protocol on Monitoring and Evaluation is tasking, requires innovation and technical expertise [13]. Deliverability, affordability, scalability, and sustainability of the study proposed was the criterion that was awarded the highest scores in the assessment of the submitted concept notes. This is because it is recognized that although these were small scale studies which were to be implemented as student's projects, they should have potential to be scaled up to national or sub national levels in the future. “Answerability and ethics” scored the next highest scores because this criteria took into consideration the feasibility of methodology of proposed and the benefits and harms that may ensue. Thus concept which studied the economics of malaria scored highest because the stakeholders emphasized that there was no data and there were major gaps in knowledge on this aspect. A multi sectoral approach was used to guide choice of the facilitators of the workshop. This approach has been used successfully by other programmes [14, 15]. Our workshop involved resource persons the federal level (NMEP) who are involved in the development and implementation of policies; academician who have good knowledge, attend scientific conferences and conduct research on malaria; implementing partners support the federal government to implement its policies and programmes, they also have data on malaria; and the NFELTP faculty who are also researchers /trainers and can support the students even after the workshop. This approach ensured that a comprehensive inventory of the research gaps in the country was obtained by drawing on the competencies and experiences of the different sectors. There were similarities between the research questions in the concept notes of the students and the inventory of operations research required in the country generated by the NMEP in 2012 [14]. In the NMEP inventory, 122 questions were formulated. This comprised of questions on integrated vector management (31.1%), case management (27.9%) and malaria in pregnancy (14.8%) and cross-cutting issues (26.2%) [14]. These similarities were particularly remarkable in the areas of “malaria in pregnancy” and “case management of malaria”. These students were assisted to develop proposals by resource persons from the NMEP. There are also request for funding by the NMEP for these studies.

The workshop had limitations. Firstly, most of the students intended to conduct the research as part of their dissertation. This implies that they have limited time, (ideally one year from initiation of the concept till defense of the dissertation) and limited funds to implement the study [15]. Hence all the concept notes received were cross sectional surveys. The limitation of this is that it may take considerable amount of time to translate the findings to interventions that can be rolled out on a large scale and impact public health practice in the country. Another limitation of the workshop is that some important knowledge gaps and research ideas may not have been identified by our technical experts. Similarly some ideas might have been included due to personal interest or excessive media interest [15]. Thus the conclusion represents the opinion of a limited group of selected people. Future workshop should involve a larger number of experts. From review of literature and to the best of our knowledge, this workshop is one of the first research prioritization workshop targeted at improving research focus of post graduate students in sub-Saharan Africa. The strength of the workshop is that it generated new and diverse research ideas. These research ideas were in the crucial areas and that will enable progress towards achieving the MDGs 4 and 5. Also these research ideas generated were in areas that are likely to attract donors. It provided an opportunity to develop collaborative links between the different stakeholders on malaria research and for students to be mentored. Finally, it enabled the training programme to develop an inventory of research problems which can be shared with subsequent cohort of students.

## Conclusion

The workshop highlighted the need to periodically expose students to areas with gaps in knowledge on major public health problems of their country. It developed a list of research priorities for the students. Similar research prioritization programmes are required in postgraduate training programmes to ensure that students and other researchers embark on studies that address the research needs of their country. Investments in workshop such as these periodically will help to track and reduce malaria disease burden in the future. It will assist funders, policy makers and researchers to make informed choices on malaria research. Similar workshops are recommended to other post graduate training programmes.

## References

[1] National Malaria Control Programme, Federal Ministry of Health (2010). Report of the national priority setting for operational research on malaria control in Nigeria: Process and outcomes.

[2] Federal Ministry of Health (2005). Federal Republic of Nigeria training manual for management of malaria in Nigeria: participants’ manual.

[3] Federal Ministry of Health (2008). A road map for malaria control in Nigeria strategic plan 2009-201 National Malaria Control Programme.

[4] World Health Organization (2013). World Malaria Report. http://www.who.int/malaria/publications/world_malaria_report_2013/en.

[5] National Population Commission (NPC) (Nigeria), National Malaria Control Programme (NMCP) (Nigeria), and ICF International (2012). Nigeria malaria indicator survey 2010.

[6] Federal Ministry of Health, National Bureau of Statistics, United Nations Population Fund, and United Nation Children's Fund (2011). Multiple indicator cluster survey, Federal Ministry of Health, National Bureau of Statistics, United Nations Population Fund and United Nation Children's Fund.

[7] Nigeria Field Epidemiology Laboratory Training Program/Nigeria Center for Disease Control (2013). Proposed national strategic plan update.

[8] Nigeria Field Epidemiology Laboratory Training Program (2013). Highlights of research accomplished in the NFELTP.

[9] Nigeria Field Epidemiology Laboratory Training Program (2011). Organization of a workshop.

[10] Snow RW, Mundia CW, Kinyoki D, Linard C, Baba ES, Adegbe E, Ozor L, Mohamed AB, Amratia P, Kabaria CW, Noor AM (2013). A description of the epidemiology of malaria to guide the planning of control in Nigeria.

[11] Olayinka AI, Agbaje AA, Alonge TO, Ekpenyong GD, Gbadegesin AS, Isiugo-Abanihe IM, Oriaku IM, Raji OA, Taiwo VO (2004). Guidelines to writing a doctoral thesis: Ibadan: Post graduate School.

[12] Malaria Programme Review (2013): Report of Malaria Programme Review (2012). National Malaria Control Programme.

[13] Gregg MB (2008). Field Epidemiology.

[14] Monitoring and Evaluation Branch, National Malaria Control Programme, Federal Ministry of Health (2013). Report of the 3rd operational research platform symposium on malaria prevention and control.

[15] Rudan I, Kapiriri L, Tomlinson M, Baliet M, Cohen B, Chopra M (2008). Evidence based priority setting for health care and research: tools to support policy in maternal, neonatal and child health in Africa. PLOS medicine.

